# Effects of Whole Body Electromyostimulation on Physical Fitness and Health in Postmenopausal Women: A Study Protocol for a Randomized Controlled Trial

**DOI:** 10.3389/fpubh.2020.00313

**Published:** 2020-07-23

**Authors:** Alvaro Pano-Rodriguez, Jose Vicente Beltran-Garrido, Vicenç Hernandez-Gonzalez, Joaquim Reverter-Masia

**Affiliations:** ^1^Research Group Human Movement, University of Lleida, Lleida, Spain; ^2^EUSES Health and Sport Sciences School, Rovira i Virgili University, Tarragona, Spain

**Keywords:** whole-body electrical muscle stimulation, whole-body electrostimulation, physical exercise, physical fitness, healthy aging, elderly, public health

## Abstract

**Background:** Age-related problems such as chronic diseases, functional limitation and dependence, reduce the quality of life in the elderly, and increase public spending in health. It has been established that physical activity plays a fundamental role in the health of the elderly. The whole body electromyostimulation (WB-EMS) could be a successful methodology as high-intensity training to improve the physical fitness of older people.

**Methods:** A minimum of 13 women between 55 and 70 years old will be randomized in two groups. The exercise with WB-EMS group (EX + WB-EMS) will conduct a resistance strength training program with superimposed WB-EMS while the exercise group (EX) will perform only resistance strength and aerobic training. Balance, strength, flexibility, agility, speed, and aerobic performance (EXERNET battery and progressive resistance test), as well as body composition, blood parameters and physical activity reporting (IPAQ-E) will be assessed to analyze the effects of whole body electromyostimulation in the physical fitness and the health in postmenopausal women.

**Discussion:** Innovative and scientifically well-designed protocols are needed to enhance the knowledge of the body's responses within this training methodology which is being used by a big quantity of population. This trial will provide evidence on the effectiveness of whole-body electromyostimulation in physical fitness and health in elderly women.

**Trial Registration:** ISRCTN15558857 registration data: 27/11/2019 (retrospectively registered).

## Background

In recent years, the aging of the world population is increasing at an accelerated rate. In 2017, world population over 60 years old was of 962 million people, which doubled the 1980 figures when there were 382 million. In 2050 the number of elderly people is expected to reach 2.1 billion (1). Two key factors influence the aging of the population. The first one is the increase of life expectancy: on average, people around the world are now living longer. The second one is the decrease of fertility rates (2). Aging is associated with a decline of physical fitness, which has a negative impact on their quality of life and increases their level of dependence (3). As a result, global concern is being generated, both for what aging means for the health of the elderly, and the increase in public spending associated with it (4).

Faced with this situation, many governments are beginning to adopt policies for the prevention, reduction and treatment of diseases derived from advanced age to promote healthy aging (5). It has been established that physical activity plays a fundamental role in the prevention and treatment of age-related problems such as chronic diseases, functional limitation and dependence, which also makes it a facilitating element of a good quality of life (6, 7). In fact, physical activity has been presented as an excellent medicine to prevent and treat various chronic diseases derived from aging (8).

Succinctly, there are several aspects related to physical fitness affected by aging whose impact can be reduced with adherence to physical activity programs. These benefits have been observed particularly in the postmenopausal woman (9), whose hormonal status enhances the risk of diverse health diseases (10). One of the most widespread consequences is sarcopenia. Sarcopenia is the major cause of falls and inability to perform typical daily life activities. It increases dependence, morbidity and death probabilities (11, 12). The decrease in strength, coupled with the balance deficit and instability in gait are the greatest risk factors for falls in older people (13). Sarcopenia has a high prevalence in postmenopausal women, leading to mobility restriction, functional impairment, physical disability and fractures (14). As a solution to this physical decline, it has been established that there is clear evidence of exercise's effectiveness as prevention and remedy against physical frailty, sarcopenia (15, 16), loss of balance and gait difficulties (17, 18). For this reason, the key components of preventing falls training include muscular strength, in addition to balance, walking displacement skills and flexibility (19).

Aging is also the biggest risk factor associated with cardiovascular diseases (20) and due to this, it has been established that age is a facilitating element of metabolic syndrome. As a result of the aging population, cardiovascular problems generate more deaths in Europe than any other cause. In many countries, it even doubles the number of deaths caused by cancer (21). The evaluation of aerobic performance can predict cardiovascular problems (22, 23). Hence, reliable and quality of evidence has established the benefit of cardiorespiratory exercise as a prevention and treatment of cardiovascular diseases (24, 25), contributing decisively to healthy aging (26). In fact, metabolic syndrome is described as an association of cardiovascular risk factors and high levels of HDL cholesterol and triglycerides (27). Exercise as an intervention for patients with metabolic syndrome leads to improved coronary artery disease risk factors, including atherogenic dyslipidemia, blood pressure, and fat metabolism (28).

Parallel to these decreases in strength and cardiorespiratory capacity, older people also experience decreasing body composition and agility with advancing age, which significantly limits their ability to perform daily activities (29). This is where exercise can play a role of great social relevance, since adherence to physical activity programs has been associated with improvements in body composition (30–32), agility (33, 34) and functional capacity (35–37).

Apart from the previously commented diseases, blood tests allow the detection of some other ones which increase with age. On one hand, aging is a risk factor for increased insulin resistance, which makes the glucose levels appear high. This plays an important role in the development of type 2 diabetes (38–40). Physical activity has shown great effectiveness in the prevention and treatment of this disease through programs aimed at the development of strength (41) and aerobic resistance (42, 43). On another hand, the prevalence of thyroid disorders increases with age and the most common thyroid disease in older individuals is hypothyroidism what can be observed in high levels of thyroid-stimulating hormone (TSH) (44). Concretely in women, it has been demonstrated that hypothyroidism is related to low physical activity levels (45).

Despite all the benefits it offers as compensation for the deterioration of physical fitness and health, physical activity has been a resource in critical disuse as a preventive and efective strategy for healthy aging (46). Mainly, sedentary behavior is primarily observed in older people. In fact, they spend 65–80% of their waking time in a sitting position (47). Therefore, to overcome sedentary lifestyles and promote adherence to physical exercise in the elderly, it will be necessary for health authorities to generate physical activity programs with an attractive profile. This programs should provide easily perceived benefits and should enjoy good social support (48).

In recent years, high-intensity training programs have been developed with older people, successfully developing increments in strength (49–53) and resistance (54, 55). Thus, it is possible that whole-body electromyostimulaiton (WB-EMS), as a training method that facilitates a high intensity of charges, could be an appropriate methodology for the exercise of this population group. Traditional local EMS is based in the application of a rectangular, biphasic and symmetrical electrical current to defined muscles by placing on them single surface electrodes. The direct electrical impulse produces muscle contraction by transcutaneous peripheral nerve stimulation (56). Recently, through some technical developments, EMS progressed to WB-EMS by using a suit in which electrodes are strategically placed on the targeted muscles. The WB-EMS devices generally allow the simultaneous activation of the muscles in thighs, arms, buttocks, abdomen, chest, lower back, upper back, wide dorsal, and two auxiliary channels of free choice. The total electrode area is 2,800 cm^2^ (57). Kemmler et al. (58) compared the effects of a WB-EMS training with those of a traditional high-intensity interval training, concluding that both programs showed the same effectiveness in improving the physical condition of sedentary men with cardio-metabolic risk. Following this line of research, the present study has raised the possibility of using this methodology as high-intensity training to improve the physical condition of older women. Our study will take into account that the WB-EMS guarantees sufficient effort in those unable or reluctant to do it on their own initiative (59).

Previous studies have analyzed the effects of WB-EMS on the health of older people, finding improvements in cardio-metabolic risk and sarcopenia (59–65). In addition, the WB-EMS has established itself as an effective method of physical conditioning, achieving improvements in VO_2_max, Aerobic Threshold, Anaerobic Threshold and Economy in the race (66), maximum isometric strength of the leg extenders, vertical jump, and handgrip strength (58, 59, 65–67).

Unfortunately, the high risk of bias of some of the previous studies leads to little evidence regarding the effectiveness of training with WB-EMS (68). So, there is a need for new studies which ensure the use of protocols compatible with a rigorous scientific methodology. The objective of this study is to analyze, from a broad and multivariable perspective, the influence of a 10-weeks WB-EMS training program on the physical condition and health of postmenopausal women using a 2-arm parallel group design with follow-up.

## Methods/Design

### Experimental Approach

The study is designed as a blinded two-arm randomized trial with parallel-groups and a follow-up. This study protocol is reported following the SPIRIT guideline for standard protocol items in interventional trials (69). The study protocol is registered in the ISRCTN registry (trial-ID: ISRCTN15558857 (70) where all the important modifications will be recorded.

**Figure 1:**
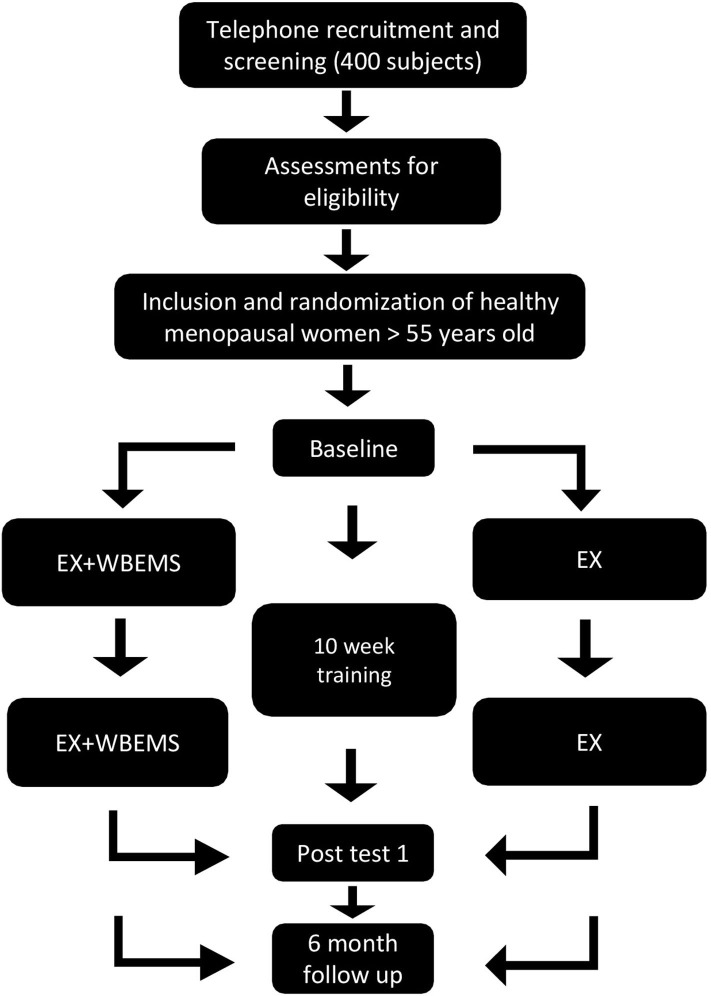
Experimental design diagram.

Participants will be blinded and distributed into two groups by a computer random number generator (71), the voluntary exercise with WB-EMS group (EX + WB-EMS) or the voluntary exercise group (EX). The EX + WB-EMS group will conduct a resistance strength training program with superimposed WB-EMS while the EX group will perform only resistance strength training. Participants will be evaluated at the beginning (baseline), at the end of the 10 weeks of intervention (post-test) and 6 months after the end of the intervention (follow-up) (see Figure 1).

The sample size was determined by an a-priori power analysis using the G^*^Power3 software for Mac (72) following the indications of Beck (73). The effect size value used was: d = 0.70 with a total sample size of 30, based on a study of WB-EMS training on hip circumference in postmenopausal women (60), with two levels for the between-subject factor (EX+WB-EMS, EX), three levels for the within-subject factor (Baseline, 10 weeks, 6 months), alpha error probability set at 0.05 and a power of 0.80. This analysis indicated a minimum total sample size of 22 participants. Considering a dropout rate of 25%, 14 participants per arm will be required. In order to check the minimum effect size to which the model will be sensitive, a sensitivity analyses with the overestimated sample size assuming a 25% of dropout rate (*n* = 28) was done. The power and alpha values used were 0.80 and 0.05, respectively. The model will be sensitive enough to detect effects as small as d = 0.60.

### Participants

The target of this recruitment is post-menopausal women between 55 and 70 years old (400 subjects) with a sedentary lifestyle at least 4 months prior to the study. They will be enrolled in the Ekke sports center in Lleida (Spain). They will be contacted by phone call to be informed about the nature of this study. All of them will be invited to attend an informational meeting where more details will be given on the benefits and possible risks that their participation in the project might entail. The subjects who show interest in their participation will be recruited, according to the exclusion criteria. They will be allocated and informed about their assigned arm by phone call by an external collaborator.

According to the shelf reporting in the IPAQ questionnaire of weekly physical activity, participants should be in a sedentary situation according to the scales provided by the Eurobarometer (74). Those who suffer from heart disease, metabolic disorders, tumor processes, or neurological disturbances will be excluded from this study. All participants will be informed of the details of the study and will sign an informed consent form before starting the investigation. The protocol of this study has been approved by the ethical committee of the Arnau of Vilanova's University Hospital, Lérida, (Spain). Participants will be informed of their voluntary participation. Taking into account the possible discomfort which WB-EMS could generate in the participants, they will be informed of their right to withdraw from the study without any prejudice or harm. The deterioration of the participant's health or any accident that could influence the correct development of the treatment will be reasons that will lead to their exclusion at any stage of the intervention. They will be also assured of their anonymity and the reporting of their views in aggregate form to protect their identities.

#### Menopause Status

Hormone assessments will be performed from fasting serum samples taken between 8:00 and 10:00 AM. Serum will be separated by centrifugation for 10 min at 2,200 × g. Systemic FSH levels will be immunoassayed using IMMULITE 2000 XPi (Siemens Healthcare Diagnostics, UK). Participants' menopause status will be determined based on the self-reported menstrual cycle.

Applying the categorization of (75) subjects will be categorized as postmenopausal if there has been no menstrual bleeding during the 6 previous months and following cut values will be applied FSH > 30 IU/L. Participants will write a self-report concerning their health problems, gynecologic status, and use of medications.

### Interventions

Both groups will train with a frequency of 2 weekly sessions. They will have 48 h of rest between sessions. To ensure the blinding of participants, the EX + WB-EMS group will train on Mondays and Thursdays while the EX group will train on Tuesdays and Fridays. Both groups will perform the same program consisting of endurance tasks and resistance strength exercises, but the EX + WB-EMS group will also implement a superimposed WB-EMS during the training. The training protocols will be supervised by two instructors graduated in Physical Activity And Sports Sciences with wide experience in WB-EMS training. Participants were asked not to make physical efforts outside the training program.

#### EX Group Intervention

The sessions will last 40 min. Participants will perform a 10-min warm-up by walking on a treadmill. Participants will be instructed to walk at the speed they normally would during a stroll in the park. Subsequently, participants will execute the resistance training protocol, which will consist of performing three multi-articular exercises involving push and pull actions (squat, deadlift, and bench press) as it is recommended (76–78).

The strength training will last 10 min divided into two blocks of 5 min. One block will consist of 10 sets of each exercise. Sets will be composed of two repetitions with 2 s of eccentric and 1 second of concentric phase (6 s in total per set). Between repetitions, the participants will have 4 s of rest.

The intensity of the resistance training will be 40% of the 1RM (77), assessed by an indirect measurement test (79). Following the line of (80) the absolute load will be increased by 5% every 2 weeks to apply the progressive overload principle. After the strength exercises, participants will perform 10 min of cardiovascular work on the treadmill, at a constant individualized speed, obtained from the talk test (81). The highest speed they can walk while talking will be estimated. The intensity of cardiovascular training will be increased by 5% every week. Finally, the participants will perform a 10 min stretching of the whole body's muscles as a cooldown.

At the end of each session, a scale will be presented (82) with a range from level 6 (No exertion at all) to 20 (Maximal exertion) in which the participants will record their extent of perceived exertion. The assessment will always end at 15 (Hard).

#### WB-EMS Group Intervention

**Figure 2:**
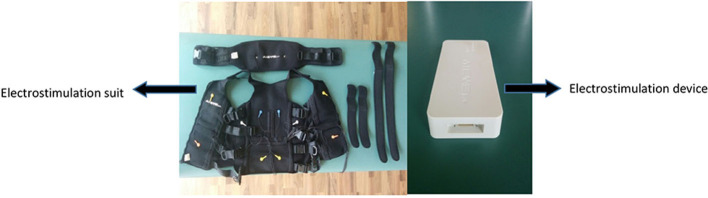
Wiemspro suit and device details.

The EX + WB-EMS group will perform the same training as EX group but with superimposed WB-EMS. A rectangular, bipolar compensated current of 6 s duration and 4 s of rest will be applied with a Wiemspro® electrostimulator (Malaga, Spain) (Figure 2). The weight of the complete kit does not reach 1.5 kg.

Since there is evidence that a minimum current frequency of 50 Hz is necessary to cause adaptations in strength training (83, 84), a frequency of 55 Hz will be applied during the strength exercises, with a 60% duty cycle (pulse width: leg, glute 350 μs, lumbar, abdominal, dorsal 300 μs, Trapeze 250 μs, chest 200 μs, arm 150 μs), 800 ms of ascent ramp, and descent ramp of 500 ms. During cardiovascular training on the treadmill, the frequency will be 7 Hz with a duty cycle of 100%.

During the WB-EMS sessions, following a similar procedure used by (60), four levels will be established on a scale from 1 to 10 to control the intensity perception of the electrical current (IPC) in the participants. The levels are from 1 to 4 (mild), from 4 to 6 (moderate), from 6 to 8 (intense), and from 8 to 10 (pain). Participants will give constant information on the IPC during the session. The first 2 weeks, training will be conducted at “moderate” IPC level to promote familiarization and adaptation to the WB-EMS. The remaining 8 weeks of the intervention, the intensity will increase to an “intense” PIC level.

### Harms

Adverse events including physical injuries will be monitored by the instructors of the intervention and the responsible staff of the assessments and documented through the facility's incident reporting process.

### Outcomes

Assessments will be carried on during the study development as it is shown in Table 1. Participants will be asked not to take any stimulants before assessments to avoid influencing the results. The data will be recorded in a spreadsheet that will be stored in an encrypted USB by an external collaborator, to guarantee the privacy of the participants. He will assess the safety and validity of the research data. A blinded statistician will have access to the final dataset of the study.

**Table 1:** Overview of the assessments schedule at baseline and follow-ups.

**Activity/Assessment**	**Study Period**				
	**Pre-study**	**Baseline**	**Intervention**	**Post-estudy**	**6 month Follow-up**
			**(10 weeks)**		
**TIME POINT**	**T-1**	**T0**	**T1**	**T2**	**T3**
Eligibility	x				
Informed consent	x				
Randomization	x				
**INTERVENTIONS**					
Exercise group (EX)		x			
Exercise + WB-EMS group (EX+WB-EMS)		x			
**ASSESSMENTS**					
Physical fitness: Balance, leg flexibility, arm flexibility, leg strength, arm strength, agility, speed, cardiovascular endurance		x		x	X
Mean velocity (m·s-1) and the mean power of exertion (w)		x		x	x
Body composition: Weight (kg), height (cm), body mass index, body fat percentage, fat mass (kg), Visceral fat (kg), lean mass (kg), Abdominal fold, Contracted arm fold, waist perimeter, hip perimeter and Sum six folds (%).		x		x	X
Blood test: Creatin Kinasa, HDL cholesterol and triglycerides, Sodium and potassium, Thyroid Stimulating Hormone (TSH), Glycohemoglobin A1c (HbA1c)		x		x	X
Lifestyl & Health behavior: Physical activity questionnaire (IPAQ): Vigorous physical activity (AFV), Moderate physical activity (AFM) or Walking		x		x	x

#### Physical Fitness

One of the evaluations of the physical performance will be carried out by the EXERNET test consisting of 8 tests modified and previously adapted from the “Senior Fitness Test Battery” and “Eurofit Testing Battery” (85).

1. Balance: “Flamingo test.” Participants will start standing, with both feet on the ground. After the signal, they will try to stand on the sole of one foot. The time the subject can stay in that posture up to a maximum of 60 s will be recorded. The test will be performed alternately, twice with each leg. The best attempt of the four will be registered.

2. Leg strength: “Chair Stand Test.” The participant will start from a sitting position with her arms crossed and the palms of her hands resting on her shoulders. The number of times she will be able to get up and sit in 30 s will be registered. The test will be performed only once.

3. Arm strength: “Arm Curl Test.” Participants will sit on a bench holding a 2.5-kg dumbbell. The maximum number of elbow's flexo-extension that the participant will be able to execute in 30 s will be registered. The test will be performed once with each arm.

4. Legs flexibility: “Chair Sit-and-Reach Test.” Participants will begin the test sitting, with one leg extended and the heel resting on the floor, while her hands will be directed toward the toes of that leg. The existing distance, positive or negative, in centimeters, between the fingers and toes will be registered. The test will be performed once with each leg.

5. Arms Flexibility: “Back Scratch Test.” The participant will place a hand over the shoulder of that same arm, and the opposite hand from the bottom up, trying to touch each other. The participant will try to touch or overlap the fingers of both hands. The distance in centimeters (positive or negative) between the fingertips of each hand will be registered. The test will be carried out twice, once with each arm.

6. Agility: “8-Foot Up-and-Go Test.” From a sitting position, the seconds that the participant will take to get up, walk to a cone located at 2.45 m, go around it, and sit down again will be registered. The test will be performed twice with at least 1-min rest between repetitions and the best result will be recorded.

7. Speed: “Brisk Walking Test.” The time taken for each participant to walk 30 m will be measured. Two repetitions will be performed with a minute of rest between them. The best of both results will be recorded.

8. Cardiovascular resistance: “6-Min Walk Test.” In a circuit of 46 meters delimited by cones, the distance in meters that each participant will be able to cover walking for 6 min will be registered.

In addition to the mentioned evaluations, a progressive resistance test (PRT) (86) will be carried out to make a more accurate assessment of the strength development. The PRT permits simultaneous direct calculations of strength (N), velocity (m·s-1). and power (w), produced with different loads, and at the same time. Taking into account that as a consequence of aging, losses in strength are observed mostly in the type II fibers (87), the mean velocity and the mean power of exertion will be assessed in this study.

Following the PRT test protocol, we will assess the mentioned variables with the execution of 6 to 8 series of 2 to 3 repetitions in squat and bench press, applying the maximum acceleration possible, alternated with rest intervals of 2–5 min. The rest period is proportional to the intensity and duration of the effort, to avoid the prediction errors caused by the accumulated fatigue. The load will be increased progressively with the series. For each magnitude of weight lifted, it is necessary to select the repetition with which the highest values of average velocity and power are reached, as this factor expresses the highest mechanical efficiency of the exercise (88). In this study, the best repetition of the best series will be recorded. Then, the load in which that best series is done in the pre-test will be used in the post-test to compare the evolution of the variables after the intervention in the post-test 1 and the follow-up.

To perform this test a lineal encoder Chronojump® (Bosco–System, Barcelona, Spain) will be used in order to detect the position of the resistance during linear movements. This data permits an estimate of the range of movement, acceleration, velocity, strength and the power produced during each action (89).

#### Body Composition

The following parameters will be measured: height, weight, body mass index (BMI), body fat percentage, fat mass, visceral fat, lean mass, abdominal fold, contracted arm perimeter, waist perimeter, hip perimeter, and sum of six folds.

Height will be determined with an accuracy of 0.10 cm with a SECA stadiometer (SECA, Hamburg, Germany). Participants will stand erect without shoes, with heels together and their head in the Frankfort horizontal plane. Body weight will be evaluated with an electronic balance with a sensitivity of 0.10 kg (Tanita BC 418 MA, Tanita Corp. Tokyo, Japan). Body mass index will be obtained using the formula: body weight / height^2^. The fat mass, visceral fat and lean mass will be estimated by bioelectrical impedance analysis (BIA) using an eight-contact electrode segmental body composition analyzer (Tanita BC-418, Tanita Corp. Tokyo, Japan).

To assess skinfolds and perimeters, a 0.50 mm sensitivity Slim Guide caliper and a measuring tape (CESCORF) will be used, respectively (90). The muscle mass will be estimated by using the formula of (91).

All measurements will be made in duplicate non-consecutively and using the average value as the final value. All women will be measured at the same time of the day for pre- and post-intervention to avoid errors due to differences in hydration. All analyses will be performed by a level I anthropometric technician certified by the International Society for the Advancement of Kineanthropometry (ISAK) as described in its Reference Manual (92).

#### Blood Test

A full analytical profile will be made to extract different parameters detailed below. The activity of Creatin Kinasa will allow analyzing the muscle damage caused by the exercise (93). High density lipoprotein (HDL) cholesterol and triglycerides will yield information on the presence of metabolic syndrome, understanding this as an association of cardiovascular risk factors (27). Glucose will be measured to analyze the presence of diabetes mellitus and know cardiovascular risk (94). The level of oxidative stress and cardiovascular risk will be known through uric acid (95). Sodium and potassium will be examined to determine if there is an adequate response to the osmotic balance in the body (96). The status of the thyroid gland will be determined by observing the levels of Thyroid Stimulating Hormone (TSH) (97). Finally, Glycohemoglobin A1c (HbA1c) will allow monitoring the evolution of sugar in the months prior to the study (98, 99). The blood tests will be made by the endocrinology department of the Arnau of Vilanova's University Hospital, Lérida, (Spain).

#### International Physical Activity Questionnaire (IPAQ-E)

The IPAQ-E consists of seven open questions in reference to the activities carried out by the elderly in the last 7 days. These questions evaluate the intensity by classifying it into Vigorous Physical Activity (VPA), Moderate Physical Activity (MPA), or Slight Intensity Activities (SPA). They also evaluated the frequency (days per week) as well as the time spent in each of these activities. The Total Physical Activity (TPA) is the sum of the VPA, the MPA, and SPA (100). They are considered VPA those that involve an intense physical effort and that entails breathing much more intensely than normal. MPA requires a physical effort that increases breathing at a somewhat more intense intensity than normal. Slight intensity activities include walking recreationally or for leisure (101). Only activities that last a minimum of 10 min will be considered for registration. The IPAQ-E will allow verifying the untrained condition of the participants since it gives information about the amount of physical activity which they practice in their daily life. At the same time, IPAQ-E will be the control tool regarding the amount of physical activity which the participants will practice in the time run between post-test and follow up.

### Statistical Procedures

After checking normality and homogeneity assumptions, the effectiveness of different training programs on quantitative dependent variables will be assessed by a 2 × 2 mixed ANOVA. Group training intervention (“EX+WB-EMS,” “EX”) will be included as between-subjects factor, improvement (“10 weeks from Baseline,” “6 months from Baseline”) will be included as the repeated within-subjects factor, and group ^*^ improvement will be included to account for the interaction effects. Whenever a significant main effect or interaction will be observed, Bonferroni's *post hoc* correction will be used to aid interpretation of these main effects or interactions, either between-subjects factor at whatever time point or within-subjects factor at whatever group (102, 103). Hedge's *g* effect size (*g*) will be calculated and interpreted as follows *g* < 0.20 = trivial, *gfrom*0.20*to*0.50 = *mild, g* from 0.50 to 0.80 = moderate and *g* > 0.80 = large (104). Whenever the data fails to meet assumptions, robust methods will be performed following procedures of (105) and the explanatory measure of effect size (ξ) will be calculated and interpreted as suggested by (106). Values of ξ = 0.10, 0.30, and 0.50 correspond to small, medium, and large effect sizes, respectively. The qualitative ordinal variables will be analyzed with a 2 × 2 rank-based ANOVA following directions from (107). Significance level will be set at α= 0.05. The statistician will be blinded to both groups during data analyses. Intention-to-treat analysis will be performed for the primary and secondary outcomes. If necessary, a multiple imputation technique will be used to handle missing data. All analyses will be performed with JASP (version 0.11.1; JASP Team (2019), University of Amsterdam, the Netherlands) and with statistical software R Core Team (2019) for Mac and WRS2 package (108).

## Discussion

The worldwide implementation of effective protocols to study the WB-EMS's effects is limited. However, it is a much-extended training methodology which is being used by thousands of people without a specific legislative control and an adequate official instructional guide for health professionals. Therefore, the present study aims to enhance the knowledge of the body's responses within this training methodology. The aim is to analyze the effect of a training program with WB-EMS on the physical condition and health of postmenopausal women. To do so, we will carry out an intervention based on the development of the physical condition through the implementation of the WB-EMS. This will allow us to carry out a subsequent multivariable analysis of the effects of this program, through evaluation techniques of different kinds. We will proceed with the analysis of blood parameters to observe the evolution of endocrine and metabolic aspects. We will carry out a physical performance test as a way to observe the differences in physical condition. With an anthropometric analysis, we will study changes in body composition and with the IPAQ we will analyze the adherence to physical exercise generated by the intervention in the participants by comparing the total activity in the week before the intervention and the total activity in the week before the follow-up. This variety of techniques for assessing physical fitness has the purpose of approaching the problem from various perspectives, which becomes the main contribution with respect to the previous literature.

To verify the effectiveness of WB-EMS, it is essential the isolation of its effects from the ones produced by voluntary contraction. In some of the previous studies, the control group received an intervention that was not equivalent to the one applied in the experimental group (58, 109–112). This procedure prevents the analysis of the WB-EMS's effect by itself. In other studies, the control group did not receive any type of intervention (64, 113), which prevents comparability between treatments. Another study compared two different types of training with WB-EMS, which only allowed the comparison of one treatment with another but prevents the analysis of the effect of the WB-EMS by itself (66). Other studies designed interventions whose control group carried out exercises of the same nature as the experimental group, but their training did not match the volume and frequency of training loads (58, 60, 114, 115). The protocol of the present study intends to carry out an experimental phase in which the interventions of both groups are completely comparable in terms of type of movements and training volume (116–118). It will allow identifying precisely the isolated effect of the WB-EMS.

The expected effects of our WB-EMS application protocol related to the development of physical fitness are based on those found by (112) when carrying out a 12-weeks training program with sedentary men. The authors observed that, except for slight improvements in some physical variables, the changes caused by the WB-EMS training were not superior to those of other exercise programs. Similar results were found by (116) in physically active women. The authors concluded that after 4 weeks of intervention, the WB-EMS training could serve as a reasonable but not superior alternative to classic training regimes. On the basis of this finding, it could be considered that the WB-EMS could be an adequate methodology to guarantee the training intensity necessary to cause positive adaptations in the organism. While it does appear to be as effective as other methodologies such as high-intensity interval training (HIIT), WB-EMS could be a good tool in order to reach the intended training intensity with the absence of abrupt movements and high resistance loads. Thus, it would be an adequate approach to postmenopausal women's physical training.

In Filipovic et al. (119), Kemmler et al. (58), and Wirtz et al. (118) the effects of WB-EMS on anthropometric parameters were analyzed without finding significant differences between the experimental group and the control group. Regarding the analysis of blood parameters, it could appear that there was some kind of significant improvement in cholesterol levels, as it happens in (109). Due to this, given the results obtained to date, the fact of finding significant changes in the blood parameters of the present study would be possible. In Kemmler et al. (113) no changes in triglycerides, glucose and cholesterol were observed after 26 weeks of training with WB-EMS. Similar results were noted in (80) given that no differences were noted in the analysis of the evolution of testosterone, cortisol, and growth hormone.

The way in which the health professional approaches the proposal of the physical activity program to which the patient is intended to adhere seems to be of great importance. It is necessary to assess correctly the needs of the person to propose appropriate exercise to their characteristics. It will also be crucial for the health professional to know how to clearly convey the need to adopt a healthy lifestyle and plan a good methodology and guidelines that will have to be followed to achieve it (120). To comply with this methodology, this study will hold theoretical lectures in which participants will be informed about the type of physical exercise they will perform during the study. They will be explained in detail the training program that we will carry out with them as well as the benefits it will entail for their health and their quality of life. It has been established that technology-based exercise programs have good adherence and may provide a sustainable means of promoting physical activity and preventing falls in older people (121). Taking these results into account, we hope to obtain satisfactory adherence to physical activity with the sample of this study.

Some advantages and limitations of this protocol deserve comments. On one hand, as the main advantage, we should mention that it ensures the assessment of the isolated WB-EMS's effects. Besides, by carrying out this multivariate assessment it is possible to evaluate postmenopausal physical fitness from various perspectives. It can be said that the study will allow other clinicians to use a more structured guide to get reproducible results. On the other hand, as limitations, it must be pointed out that the selection of the participants is carried out from a voluntary population from a big sports center. This procedure could derivate in a selection bias. This must be considered in the generalization of the results to other health systems.

In summary, this trial will provide insight into the effect of WB-EMS in postmenopausal women, after a 10-weeks intervention. Practitioners, exercise therapists, and instructors will be provided with a feasible, validated WB-EMS training program whose effect on physical fitness and health is scientifically assessed. Finally, the results of the current trial may help to develop further theories and models explaining WB-EMS training effects in general and particularly with older adults.

## Ethics Statement

The protocol of this study has been approved by the ethical committee of the Arnau of Vilanova's University Hospital, Lérida, (Spain). Project reference: CEIC-1701. All participants will be informed of the details of the study and will sign an informed consent form before starting the investigation.

## Author's Note

Research Group Human Movement, University of Lleida, Av. de l'Estudi General, n.4 E-25001Lleida, Spain. number: +34649546894. Email: alvaro.depano@udl.cat.

## Author Contributions

AP-R and JR-M conceived and designed the protocol and conducted the article searches. AP-R and VH-G drafted the manuscript. JB-G projected the analytic plan and interpretation. All authors read and approved the final manuscript.

## Conflict of Interest

The authors declare that the research was conducted in the absence of any commercial or financial relationships that could be construed as a potential conflict of interest.

## Abbreviations

BMI: Body mass index
EX group: Exercise group
EX + WB-EMS: Exercise with whole-body electromyostimulation
IPAQ-E: International *physical activity questionnaire*
IPC: Intensity perception electrical current
MPA: Moderate physical activity
PRT: Progressive resistance test
1RM: One-repetition maximum
SPA: Slight intensity activities
TPA: Total physical activity
VO2max: maximum oxygen consumption
VPA: Vigorous physical activity
USB: universal serial bus
WB-EMS: Whole-body electromyostimulation.
